# The effects of seawater temperature-induced coral bleaching on the aragonite structure and material properties of massive *Porites lutea* coral skeletons

**DOI:** 10.1007/s00338-025-02735-5

**Published:** 2025-08-29

**Authors:** Alice Sinclair, Susan Fitzer, Samantha Greeves, Kirsty Penkman, Chalermrat Sangmanee, Nicola Allison

**Affiliations:** 1 School of Earth and Environmental Sciences, University of St Andrews, St Andrews, KY16 9TS UK; 2 Scottish Oceans Institute, University of St Andrews, St Andrews, KY16 8LB UK; 3 Faculty of Natural Sciences, Institute of Aquaculture, University of Stirling, Stirling, FK9 4LA UK; 4 Department of Chemistry, University of York, York, UK; 5 Department of Marine and Coastal Resources, Ministry of Natural Resources and Environment, Bangkok, Thailand

**Keywords:** Coral bleaching, Material properties, Skeleton, Raman spectroscopy, Organic matrix

## Abstract

The coral skeletons that contribute to tropical reef structures are biominerals, composed of inorganic aragonite and organic biomolecules. The biomolecules influence the aragonite structure and material properties of the skeleton. We collected massive *Porites lutea* skeletons from Phuket, Thailand, in 1991, approximately one month into a temperature-induced bleaching event. Some specimens had expelled their Symbiodiniaceae in response to the increased water temperatures (bleached), while other corals appeared unaffected (unbleached). We investigate the effect of Symbiodiniaceae loss on the amino acid composition, aragonite structure, and Vickers hardness of the coral skeletons. We observe no significant difference in the amino acid content or composition of the outermost 1 mm of skeleton (representing 0.5 to 2 months growth) between bleached and unbleached specimens. The full width half maximum of the Raman spectrum ʋ_1_ band, an indicator of disorder around the CO_3_ group in the aragonite lattice, varies significantly between some corals in the outermost 200 µm of skeleton, but these differences are not attributable to the bleaching status of the coral colonies. Similarly, Vickers hardness varies significantly between some colonies, but this is not related to coral bleaching. This is a positive finding, suggesting that bleaching, from which corals recover, does not adversely affect the coral skeletal structure.

**Supplementary Information:**

The online version contains supplementary material available at 10.1007/s00338-025-02735-5.

## Introduction

Reef-building scleractinian corals secrete hard skeletons, transforming soft ocean floors into complex structures, deemed the most biodiverse marine ecosystem (Hoegh-Guldberg 2015; Tambutté et al. 2011). Coral reefs support critical ecosystem services such as fisheries, tourism, and coastal protection, offering significant socioeconomic benefits (Hoegh-Guldberg 2015). Rising ocean temperatures threaten corals by disrupting the symbiotic relationship between the coral polyps and the Symbiodiniaceae hosted in the coral cells (Lesser 2006). When sea surface temperatures (SST) rise ~ 1–3 °C above historical maxima (Coles and Brown 2003), the algae’s photosynthetic efficiency decreases (Iglesias-Prieto et al. 1992), ultimately resulting in algal expulsion. This process, known as coral bleaching, places corals at a heightened risk of starvation and impacts several vital internal processes (Cróquer and Weil 2009; Eakin et al. 2019; Hughes et al. 2019), including calcification. Global temperature rise associated with increased anthropogenic CO_2_ emissions (IPCC 2022) has increased the frequency and severity of bleaching events (DeCarlo 2020; Babcock et al 2020). Understanding the full impact of coral bleaching on biomineralisation is essential for predicting the effects of climate change on coral reefs.

Coral skeletons are biominerals, composed of aragonite and organic biomolecules, including polysaccharides, proteins, and lipids (Cuif et al. 2003, 2004; Fukuda et al. 2003; Patton et al. 1977). Biomolecules influence the nucleation, precipitation rate, morphology, and structure of aragonite precipitated in vitro (Kellock et al. 2020, 2022; Nahi et al. 2023; Castillo Alvarez et al. 2024) and increase the hardness of calcite precipitated in vitro (Kim et al. 2016). For these reasons, the coral skeletal organic matrix is inferred to control biomineralisation and to improve the material properties of the skeleton. The amino acid, protein, sugar and lipid compositions of coral skeletons vary between genera (Cuif et al. 1999a,b; Watanabe et al. 2003; Farre et al 2010). Some amino acids and monosaccharides are significantly different between skeletons from corals which either harbour or do not contain Symbiodiniaceae (Cuif et al. 1999b), suggesting that nutritional lifestyle influences the amino acids integrated within coral skeletons (Muscatine et al. 2005; Ferrier-Pages et al. 2021).

In this research, we investigate the effect of coral bleaching on the amino acid composition of coral skeletons, on skeletal aragonite structure, and on the Vickers hardness of the skeletons. We study a series of massive *Porites lutea* coral skeletons collected during a heat-induced bleaching event in Thailand in 1991 when some of the collected individuals were bleached and others appeared unaffected (Allison et al. 1996). We use reverse phase high-performance liquid chromatography (RP-HPLC) to analyse the amino acid composition of the outermost 1 mm of the skeletons, reflecting the aragonite deposited shortly before collection. We use Raman spectroscopy to estimate disorder around the CO_3_ group in the aragonite lattice (Bischoff et al. 1985; DeCarlo et al. 2017) and microindentation to measure the skeletal hardness, a measure of resilience to plastic deformation (Fitzer et al. 2019). We compare these metrics for bleached and unbleached corals. Coral bleaching events are expected to increase in frequency and severity as global warming continues to rise towards a 1.5 °C increase above pre-industrial levels (IPCC 2022). The present study resolves the impacts of such bleaching events on the structure and physical integrity of coral skeletons calcified under heat stress.

## Methods

### Coral collection and sampling

Sample collection is described in Allison et al. (1996), and the specimens analysed here are summarised in Table 1. In brief, coral skeletons were collected from a reef on the SE coast of Phuket Island, Thailand (GPS coordinates 7.806, 98.411). The site consisted of a 100–150 m intertidal reef with a steep sloping reef front which dropped to muddy sediments at up to 6 m below the mean low water mark. The reef front was dominated by large (up to ~ 4 m diameter) massive *Porites* spp. colonies, typically with a hummocky morphology. Coral bleaching at this site and the surrounding reefs was observed from early June until the end of July 1991. Mean monthly sea surface temperatures usually ranged from ~ 27.9 °C (January) to 29.4 °C (May) but were up to 1 °C above normal from December 1990 onwards, and in May and June 1991, temperatures reached > 30 °C (Allison et al. 1996). Some corals at the site became completely bleached while others, of the same genera and at the same depth, retained a typical dark brown pigmentation. Six unbleached and four bleached specimens were collected from 1 to 3 m below the mean low water levels over an 11 day period in July 1991.

**Table 1:** Summary of coral specimens analysed in the present study. The total skeletal amino acid concentrations, mean Raman spectrum ʋ_1_ band FWHM (± 1σ) and mean Vickers hardness (± 1σ) were determined in the present study. Skeletal extension rates and coral tissue [chlorophyll a] are reproduced from Allison et al. (1996). Skeletal extension rates were estimated from the fluorescent banding in the corals and record the distance along the maximum growth axis between the start of the final bright fluorescent band in the skeleton (deposited in approximately November 1990) and the outer growth surface of the coral, in the corals collected in July 1991, and between the start of the same bright fluorescent band and the alizarin red S stain line deposited in July 1991 in the corals collected in 1992. [Chlorophyll a] (Chl a) was measured in five replicate tissue samples in each colony and values represent mean ± 1σ. n.d. = not determined

Sample	Year collected	Skeletal linear extension (mm)	Chl a (mg cm^−2^)	Total skeletal amino acid (pmol mg^−1^)	Mean Raman ʋ_1_ band FWHM (cm^−1^)	Mean Vickers Hardness (GPa)
*Unbleached*						
PB1	1991	9.2	17.3 ± 2.9	1313	4.07 ± 0.03	3.40 ± 0.36
PB2	1991	13.0	n.d	1428	4.12 ± 0.04	n.d
PB3	1991	18.8	11.1 ± 1.0	1349	4.12 ± 0.04	2.94 ± 0.39
PB5	1991	10.5	7.9 ± 2.2	1550	4.08 ± 0.07	3.23 ± 0.49
PB6	1991	7.6	18.9 ± 2.4	1516	4.10 ± 0.03	3.07 ± 0.46
PB9	1991	10.2	19.3 ± 0.8	1885	4.13 ± 0.07	3.07 ± 0.54
PB2NEW	1992	12.0	n.d	n.d	4.08 ± 0.03	n.d
*Bleached*						
PB4	1991	5.0	0 ± 0	2309	n.d	n.d
PB7	1991	4.5	0 ± 0	1538	4.08 ± 0.05	3.32 ± 0.37
PB8	1991	5.0	0 ± 0	1597	4.13 ± 0.04	3.37 ± 0.44
PB12	1991	11.0	0 ± 0	2030	4.11 ± 0.07	3.09 ± 0.49
PB10	1992	5.0	n.d	n.d	4.11 ± 0.03	n.d
PB11	1992	8.0	n.d	n.d	4.08 ± 0.02	n.d

Prominent knobs of each colony, up to ~ 30 cm in diameter, were removed using a hammer and chisel. Three weeks before collection, 5 of the unbleached and 4 of the bleached corals were stained with alizarin red S (Allison et al. 1996). Three additional skeletons were collected in July 1992 of which 2 were bleached and 1 was unbleached in July 1991. These specimens were also stained with alizarin red S in July 1991. All skeletons incorporated stain indicating that some calcification occurred, even in bleached specimens. After collection, all the skeletons were submerged in 3–4% sodium hypochlorite for 48 h, rinsed in tap water and dried. Analysis of the [chlorophyll a] of the coral tissues and underlying skeleton indicated that no chlorophyll a was attributed to Symbiodiniaceae in the bleached corals (Allison et al. 1996, summarised in Table 1). Corals were identified as *Porites lutea* based on corallite morphology (Veron 1986).

Upon return to the UK, the skeletons were sawn perpendicular to the skeleton surface to produce a slice that spanned the maximum growth axis of each skeleton, i.e. where skeletal extension was most rapid. Slices were photographed under ultraviolet light to record fluorescent banding patterns. Bright and dull fluorescent bands are deposited approximately annually in corals at this site, with bright band accretion beginning in approximately November (Scoffin et al. 1992). Slices were sampled for this study in 2023. For skeletal amino acid analysis, the outermost 1 mm of skeleton was removed from the top of the maximum growth axis of the corals collected in July 1991 using a drill with a 0.5 mm bit. In the ~ 9-month period before collection, the corals extended their skeletons by 8–19 mm in the unbleached specimens, and by 5–11 mm in the bleached specimens (Table 1) so this outermost 1 mm, typically represents ~ 0.5 to 2 months of growth. Polished mounts were produced from all of the coral skeletons, with the exception of PB4 for which skeleton was limited. For the mounts, small blocks were cut from the outermost section of each slice at the top of the maximum growth axis. Each block was approximately 10 × 5 mm in dimension at the skeleton surface and extended about 15 mm into the coral skeleton, i.e. representing the last 15 mm of skeletal extension before the skeletons were sampled. Blocks were mounted in epoxy resin (Struers Epofix), ground using silicon carbide papers (1200 and 2400) and polished with 3 and 0.25 µm diamond suspension.

### Amino acid analysis

The intra-crystalline amino acids were analysed following the method of Tomiak et al. (2013). All reagents are analytical grade unless otherwise reported. In brief, < 20 mg of powdered skeletal sample (< 100 μm) was accurately weighed into a plastic microcentrifuge tube and bleached using 50 μL 12% sodium hypochlorite (NaOCl) per mg to isolate the intra-crystalline fraction of amino acids. Removal of the chemical bleach was carried out after 48 h using sequential rinsing with deionised water and methanol. After chemical bleach removal, the samples were left to air-dry. Once dry, < 10 mg was accurately weighed into a 2-mL sterile glass vial (Wheaton) and 20 μL/mg 7 M HCl was added. After a flush with nitrogen, the vials were heated at 110 °C for 24 h. This process hydrolyses the peptide bonds in the samples, allowing for the individual amino acids to be analysed. Upon removal from the oven, samples were dried in a centrifugal evaporator overnight before being rehydrated and analysed using reverse phase HPLC with fluorescence detection, following a modified method of Kaufman and Manley (1998). This enables quantification of L and D isomers of 12 amino acids. Asparagine and glutamine undergo deamination during the sample preparation process, so they contribute to observed concentrations of aspartic acid and glutamic acid. We therefore report aspartic acid/asparagine as Asx and glutamic acid/glutamine as Glx. The remaining quantified amino acids are serine, L-threonine, glycine (Gly), L-arginine, alanine, valine, phenylalanine, leucine, isoleucine, and L-histidine. All samples were run in duplicate alongside standards and blanks. The free amino acids of the skeletons were also determined (without hydrolysis) and were always < 5% of the values observed in the hydrolysed samples, indicating that little degradation of the skeletal proteins occurred between coral sampling (in 1991 and 1992) and amino acid analysis (in 2024). Analyses of replicate drilled coral powders (*n* = 5) indicates that the standard deviation (1σ) of repeat analyses is ± 125, 34, 51 and 13 pmol mg^−1^ for [total amino acid], [Asx], [Gly] and [Glx], respectively, equivalent to coefficients of variation of 8%, 4%, 20% and 7%, respectively (Kellock et al. 2020). For the remaining amino acids, standard deviation (1σ) of [amino acid] of replicate analyses were always < 12 pmol mg^−1^. The contribution of each amino acid to the total amino acid was calculated as [amino acid]/[total amino acid], with both quantities in pmol mg^−1^. Inclusion of alizarin red S stain in the skeleton does not affect the contribution of different amino acid groups to intra-crystalline protein (Kellock et al. 2020).

### Raman spectroscopy

**Fig. 1:**
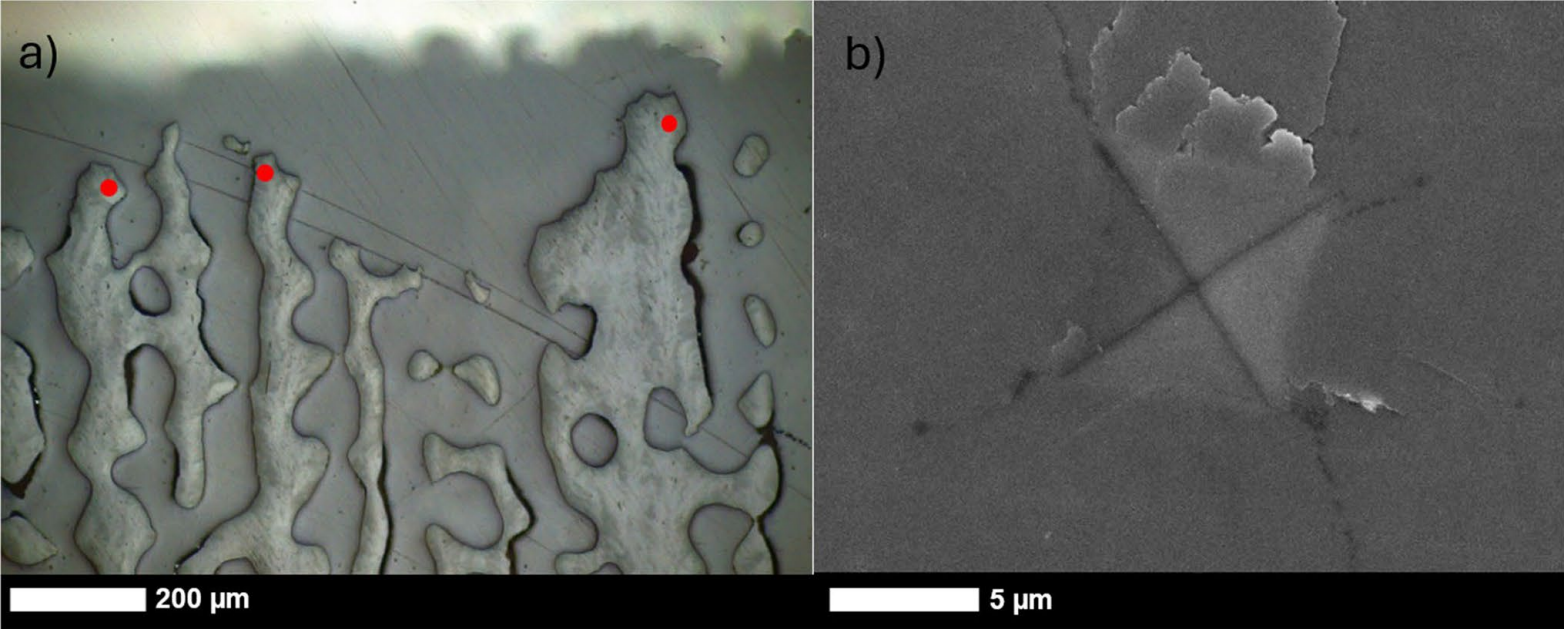
**a** Reflected light micrograph of the outermost section of a coral mount indicating sites of Raman analyses (red dots) and **b** scanning electron micrograph of a microindentor scar made on a polished coral skeleton mount using a mass of 25 g for 10 s

Raman spectra were collected using a Renishaw In-Via Qontor Raman Microscope with a 50 × objective and a NIR 785 nm solid state laser operating at 5% full power with a 1200 mm^−1^ grating and a spectral resolution of ~ 1 cm^−1^. The laser was focused to ~ 10 × 1 µm and was orientated so that the longest dimension was parallel to the growth direction of the skeleton (Fig. 1). The laser spot was targeted approximately midway between the centre and edge of each spine. Analyses were not sited on early mineralisation zones (EMZ, also called centres of calcification) which appear as dark spots or lines in reflected light imaging of polished mounts of coral skeletons (Wells 1956). These areas were avoided as they exhibit different crystal morphology (Cuif and Dauphin 2005) and are enriched in organic material compared to the bulk of skeletal material (Cuif et al. 2003).

For each analysis, 10 acquisitions of 1.5 s each were collected between 100 and 1311 cm^−1^ at the same spot and summed to provide a final spectrum. The cosmic ray removal function was enabled to remove any spurious peaks. The full width half maxima (FWHM) of the ʋ_1_ band of each spectrum was estimated by fitting the ʋ_1_ peak with a Voight fit between 1060 and 1120 cm^−1^ in OriginLabs peak processing software. The CaCO_3_ Raman ν_1_ band reflects symmetric C–O stretching of the planar carbonate group (Bischoff et al. 1985) and an increase in the FWHM of this band indicates enhanced disorder around this group in the CaCO_3_ lattice. Measured FWHM were corrected to true FWHM using the instrument spectral resolution (Nasdala et al. 2001).

Approximately 40 Raman spectra were collected from the outermost 200 µm of each of the skeletons retrieved in 1991 (Fig. 1a). This 200 µm of skeletal extension, typically represents ~ 3–12 days of growth based on estimates of linear extension rates in the colonies over the 9-month period preceding skeleton collection (Allison et al. 1996). In addition, spectra were collected along the alizarin red S stain line in the coral skeletons retrieved in 1992. This skeleton represents material also deposited in July 1991, when the skeletons were stained. Alizarin red S incorporation in coral skeletons has no significant effect on aragonite Raman spectrum ʋ1 band full width half maxima (FWHM, Allison et al. 2024). We collected Raman spectra on sections of the mount containing only epoxy resin and no skeleton to determine the effect of epoxy resin contamination on the spectra of coral aragonite. Spectra from the epoxy resin display a large Raman band at ~ 1113 cm^−1^ and a smaller band at ~ 1086 cm^−1^, of approximately one third of the intensity of the ~ 1113 cm^−1^ band (Supplementary Figure S1). This latter band potentially interferes with the aragonite ʋ_1_ peak at 1086–1087 cm^−1^ (Supplementary Figure S1). We screened all the aragonite Raman spectra for bands at 1113 cm^−1^, indicative of epoxy resin contamination of the analytical volume, and assume that any band at 1113 cm^−1^ can be used to estimate the intensity of the epoxy resin band at 1086 cm^−1^. The average intensity of the 1113 cm^−1^ band in coral skeleton analyses is ~ 960 counts above background, suggesting an epoxy resin contribution to the 1086 cm^−1^ band of ~ 330 counts. The 1086 cm^−1^ band in coral analyses has a typical intensity of 100,000 counts above background, and we consider the epoxy resin contribution to this peak to be insignificant. We observe no significant relationship between the FWHM of the aragonite 1086 cm^−1^ band and the intensity of the 1113 cm^−1^ band above background (univariate general linear model F _(1,250)_ = 0.17, *p* = 0.68).

Raman spectroscopy data were collected over four different days. The spectrometer was calibrated using the 520 cm^−1^ vibrational model of a Si standard embedded within the instrument. To track any drift in the instrument, a synthetic aragonite powder precipitated in vitro at Ω_Ar_ = 11 (Allison et al. 2024) was analysed at least 10 times before and after each coral skeleton. This in house reference material has a similar FWHM to the coral skeletons and a typical variance of ± 0.05 cm^−1^ (1 s, Allison et al. 2024). We rejected any coral analyses if the mean FWHM of this reference material changed by > 0.04 cm^−1^ between bracketed analyses. We observe small variations (up to 0.1 cm^−1^) in the mean FWHM of the reference material between days, and to correct for this, we scale the FWHM of the coral skeletons to the FWHM of the synthetic aragonite, assuming that the reference material has a FWHM = 4.32 cm^−1^.

### Vickers hardness

The hardness of biominerals is a measure of their resistance to deformation (Fitzer et al. 2019) and can be tested using indentation techniques. Coral hardness can be measured using microindentation and nanoindentation techniques (Hamza et al. 2013; Moynihan et al. 2021). Here, we use a Vickers LM 248AT microindentor to imprint a 25 g mass pyramid diamond for 10 s into the polished mounts. This resulted in deformation of the surface creating a diamond shaped scar typically ~ 10 × 10 µm in diagonal dimension (Fig. 1b). Coral skeletons are principally composed of fasciculi, bundles of acicular crystals radiating from the EMZ. The crystals are typically ~ 1 × 1 µm x ~ tens of µm long and are themselves composed of nanograins (Cuif et al. 2004; Tan et al. 2023). Organic materials and minor elements are heterogeneously distributed along the crystals at the micron scale (Cuif et al. 2005; Meibom et al. 2004). Our microindentation analyses averages hardness over multiple crystals rather than within crystals (nanoindentation).

Analyses were performed on 8 of the mounts made from coral collected in 1991. Between 26 and 36 microindentations were performed on the top 200 µm of the skeleton. As for the Raman spectroscopy analyses, indentations were sited approximately midway between the EMZ and the trabecula edge. So hardness was only measured on fasciculi. Coral skeletal hardness can vary based on skeletal orientation (Moynihan et al. 2022), and this methodology ensured that crystal orientation was similar in all analyses. After indentation, the mounts were gold coated, and the indent scars were photographed by scanning electron microscopy with a JEOL JSM-IT200 using a current of 40 nA and an accelerating voltage of 15 keV (Fig. 1b). As in other coral studies (Pasquini et al. 2015), we observed disturbance of the sample surface adjacent to many of the indents (Figure S2). We interpret these to show flaking of small patches of aragonite crystals from the mount surface. As indents were sited between the EMZ and the trabecula edge, it is likely that the fasciculi crystals are running approximately parallel to the sample surface in these areas. We conclude that flaking indicates the detachment of sections of crystals from the mount surface. We categorised the scars based on the level of flaking (Figure S2), grading them from 1 (high degree of flaking that obscures the dimensions of the indent) to 4 (no flaking). We discarded any images where flaking obscured the dimensions of the indent or where cracks propagated from the indent to the edge of the coral trabecula. For the remaining images, we measured the diagonal dimensions of the indents using ImageJ and calculated the indent surface area. This filtered dataset contained 21–33 indents from each coral colony and a total of 65 indents from bleached specimens and 145 indents from unbleached specimens. Indent surface area was converted to Vickers hardness as in Fitzer et al. (2019).

## Results

### Skeletal amino acid compositions of bleached and unbleached corals

**Fig. 2:**
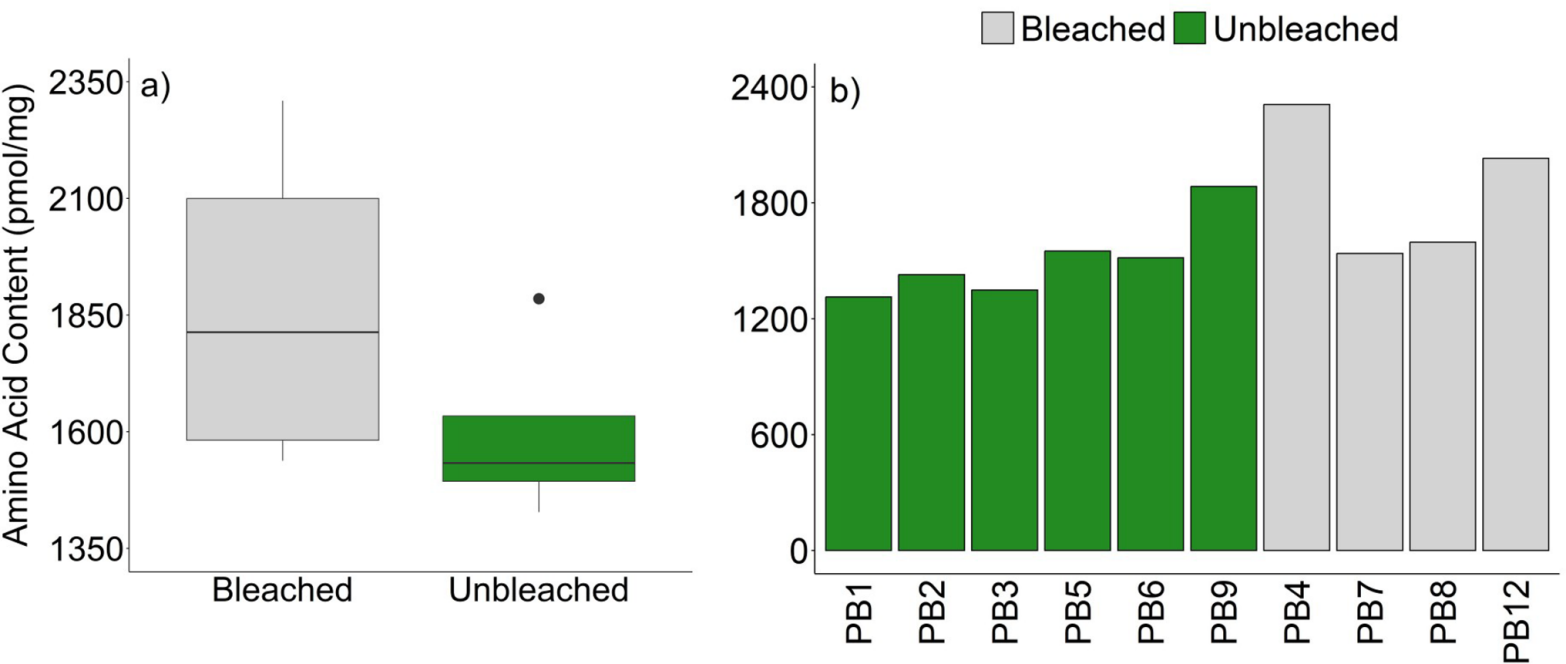
**a** Boxplot comparing total amino acid content of the outermost 1 mm of skeletons which either lost (bleached) or did not lose (unbleached) Symbiodiniaceae during the thermal bleaching event. The boxes represent the interquartile range (25th to 75th percentile) and whiskers indicate the 10th and 90th percentiles. One outlier appears above the 90th percentile in the unbleached corals. **b** Bar chart illustrating total amino acid contents across bleached and unbleached coral skeletons (*n* = 10)

The mean total amino acid concentration of each skeleton is summarised in Table 1 and Fig. 2, while concentrations of each amino acid are summarised in Supplementary Table 1. We compare the amino acid compositions of the outermost 1 mm of the skeletons collected in 1991 of the unbleached (*n* = 6) and bleached (*n* = 4) corals using a Mann–Whitney U test for equal medians, selected due to the small sample size. We observe no significant difference in the concentrations of skeletal total amino acids (*p* = 0.07) or in the contribution of each amino acid to total skeletal amino acid between the bleached and unbleached corals (Supplementary Table S2).

### Raman spectroscopy of bleached and unbleached corals

**Fig. 3:**
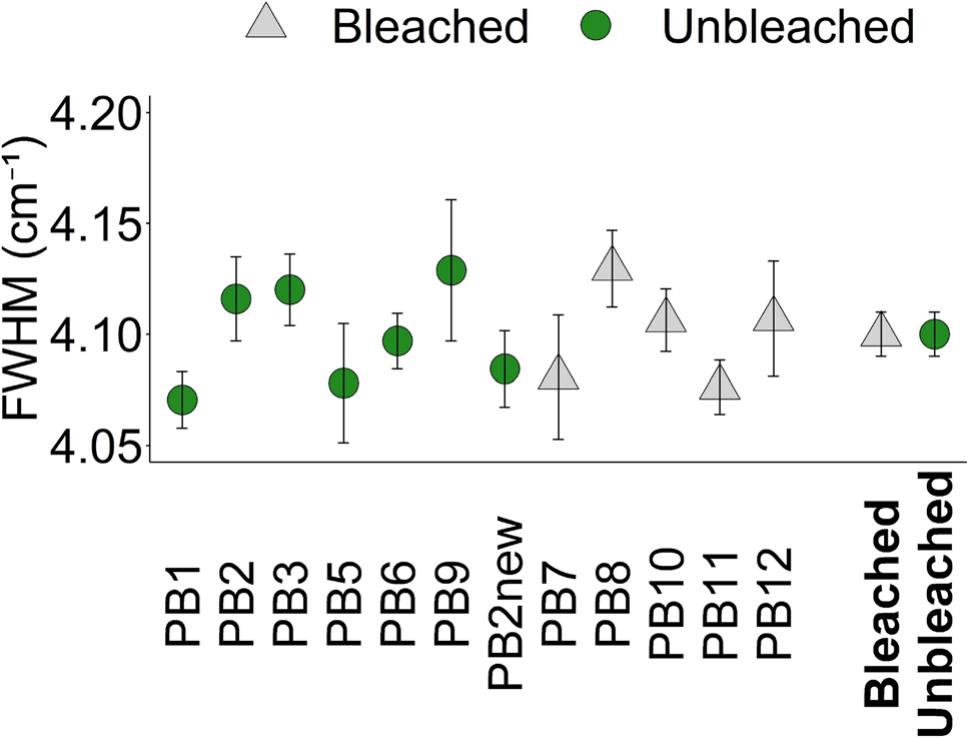
Mean FWHM of the aragonite Raman spectrum ʋ_1_ peak in the outermost 200 µm of the coral skeletons. Error bars represent 95% confidence intervals calculated across 15–34 repeat analyses per sample. The mean FWHM for grouped bleached and unbleached corals is shown in bold

All Raman spectra are confirmative of aragonite, exhibiting lattice mode peaks at 153 cm^−1^ and 206 cm^−1^ (DeCarlo 2018), a doublet ʋ_4_ peak at ~ 705 cm^−1^ (Urmos et al. 1991), and a large ʋ_1_ peak at ~ 1086 cm^−1^. The ʋ_1_ band FWHM data are detailed in Supplementary Table S3. These populations are normally distributed within each coral skeleton (Shapiro–Wilk test for normality, *p* > 0.05, *n* = 15–34, Supplementary Table S4), and we use a one-way ANOVA followed by Tukey’s pairwise comparison to test for significant differences in the FWHM between the individual skeletons. We observe some significant differences between coral skeletons (Supplementary Table S5, F_(11,246)_ = 3.91, *p* = 3.17 × 10^−5^) but variations are small (≤ 0.06 cm^−1^, Table 1, Fig. 3). To test the effect of bleaching on skeletal aragonite structure, we pool all the analyses from the bleached (*n* = 107) and unbleached (*n* = 151) corals. We observe no significant difference in the FWHM using an independent t test (T_(256)_ = 0.60, *p* = 0.55, Fig. 3).

**Fig. 4:**
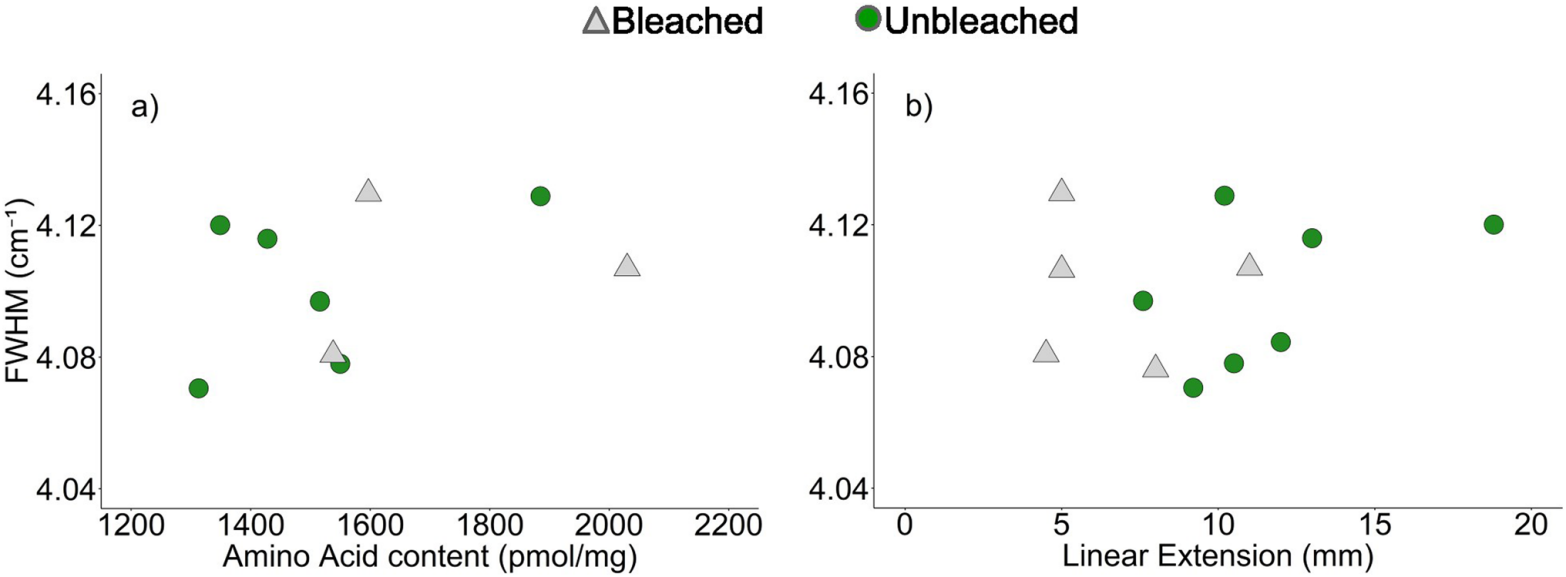
Mean Raman ʋ_1_ band FWHM for bleached and unbleached coral skeletons as a function of **a** skeletal total amino acid content and **b** skeletal linear extension (mm) over the ~ 9-month period preceding the bleaching event

We use a multivariate general linear model to test linear extension and amino acid content as additional contributors to variation in Raman ʋ_1_ band FWHM between skeletons. This model uses mean FWHM, skeletal total amino acid and linear extension for each skeleton. Initial results showed no difference in the relationship between FWHM and the predictor variables for bleached versus unbleached corals, thus subsequent analyses were conducted across the entire dataset. No significant relationship is found between Raman ʋ_1_ band FWHM and either linear extension rate or amino acid content across coral skeletons (Fig. 4, Table 2).

**Table 2:** Adjusted coefficients of determination (r^2^) and p values for relationships between Raman aragonite spectra ʋ_1_ peak FWHM, skeletal amino acid content of the outermost 1 mm of skeleton and skeletal linear extension over the ~ 9-month period preceding the bleaching event

	r^2^	*p*
Amino acid content (pmol/mg)	0.0032	0.36 (n = 9)
Linear extension (mm)	0.052	0.52 (n = 12)
Multiple linear regression (both predictor variables)	0.012	0.44 (n = 9)

Bleached and unbleached corals are pooled together for this analysis

### Vickers hardness of bleached and unbleached corals

**Fig. 5:**
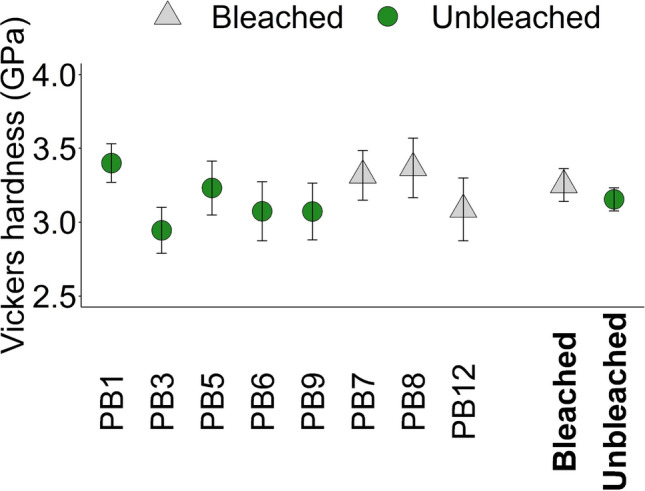
Mean Vickers hardness of five unbleached and three bleached coral skeletons, with error bars representing 95% confidence intervals calculated across 21–33 coral scars per sample (total *n* = 210). The mean Vickers hardness for pooled bleached (B) and unbleached (UB) corals is additionally shown in bold

Mean Vickers hardness of individual corals and of pooled bleached and unbleached corals are summarised in Fig. 5 and Supplementary Table S6. The populations of indent surface areas are normally distributed within each coral (Shapiro–Wilk test for normality, *p* > 0.05, *n* = 21–33, Supplementary Table S4), and we use a one-way ANOVA, followed by Tukey’s pairwise comparison to test for significant differences in hardness between the 8 coral skeletons analysed. Significant differences in hardness are observed between some skeletons, i.e. PB3 is significantly less hard than PB1 and PB8 (F _(7,202)_ = 3.58, *p* = 0.0012, Supplementary Table S7). Hardness does not differ significantly between pooled bleached and unbleached skeletons (T_(130.06)_ = 1.43, *p* = 0.16).

We categorised the scars into different groups based on the amount of flaking of skeleton from the locality of the indents (on a scale of 1 to 4), and we assessed whether scar types were associated with bleaching status. For this analysis, we calculate the contributions of each scar type to the dataset in each skeleton and use a Mann–Whitney U test for equal medians to compare the contribution of each scar type between the bleached and unbleached samples. We categorise a significantly higher proportion of scars in the pooled bleached corals as type 1 compared to the unbleached corals (Supplementary Table S8). This indicates that a higher proportion of the indents in the bleached corals result in flaking around the scar which obscures the dimensions of the indent.

**Fig. 6:**
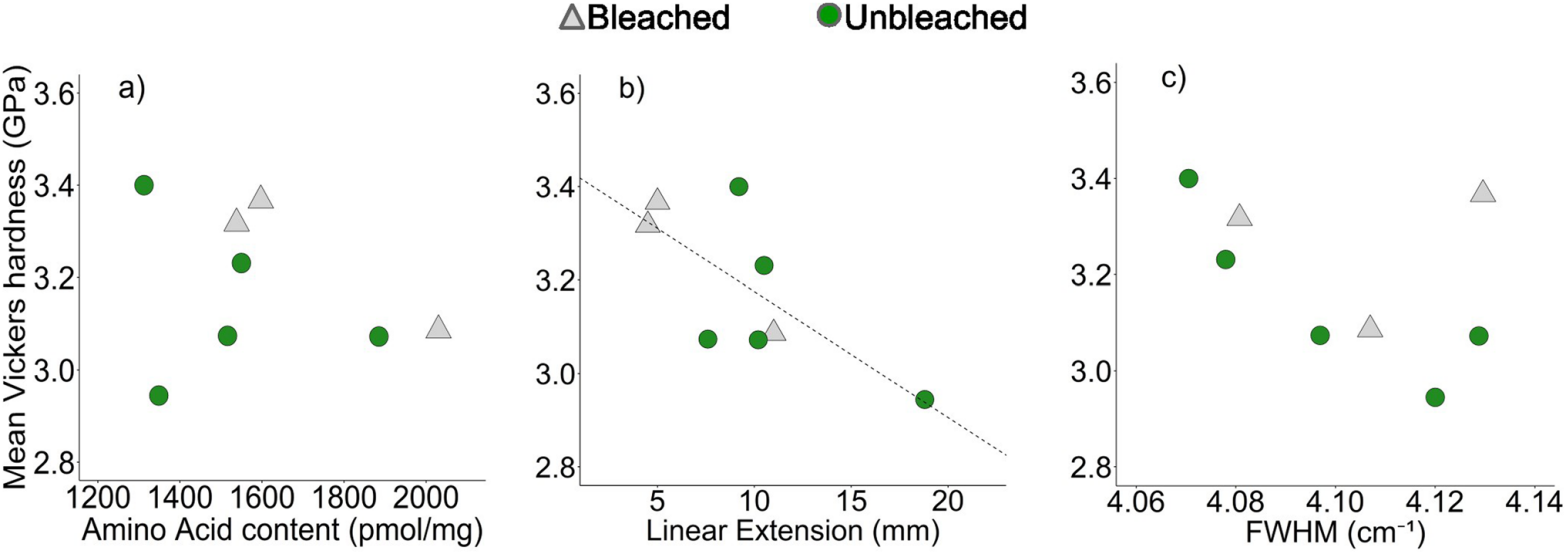
Mean Vickers hardness for 8 coral skeletons (3 bleached, 5 unbleached) as a function of **a** skeletal amino acid content in the outermost 1 mm of skeleton, **b** skeletal linear extension over the ~ 9-month period preceding the bleaching event and **c** aragonite Raman ʋ_1_ band FWHM (cm^−1^). The black dashed line shows a statistically significant linear regression

We use a multivariate general linear model to test if linear extension, skeletal amino acid content and/or Raman ʋ_1_ band FWHM contribute to variations in skeletal hardness. Initial results showed no difference in the relationship between hardness and the predictor variables for bleached versus unbleached corals and we therefore pool all the corals for subsequent analyses. We observe a significant inverse relationship between skeletal hardness and linear extension but all other relationships are insignificant (Table 3, Fig. 6).

**Table 3:** Adjusted coefficients of determination (r^2^) and p values for relationships between mean Vickers hardness, skeletal amino acid content, skeletal linear extension and Raman aragonite spectra ʋ_1_ peak FWHM

	r^2^	*p*
Amino acid content	0.080	0.51 (n = 8)
Linear extension	0.45	**0.041 (n = 8)**
FWHM	0.096	0.23 (n = 8)
Multiple linear regression (all predictor variables)	0.46	0.16 (n = 8)

Bleached and unbleached corals are pooled together for this analysis

## Discussion

### Effect of bleaching on skeletal amino acid composition

We find no significant differences in the skeletal amino acid content (Fig. 2) or composition (Table S1) of the outermost 1 mm of skeleton of *Porites lutea* corals which bleached or did not bleach during the high seawater temperature event.

Previous studies indicate that skeletal amino acids are influenced by the presence of Symbiodiniaceae (Cuif et al. 1999b) and by environmental factors e.g. seawater temperature and seawater pCO_2_ (Kellock et al. 2020; Allison et al. 2024). Increasing seawater temperature, below the bleaching threshold, significantly increased the skeletal concentrations of total amino acid, Asx, Glx, glycine and serine in cultured massive *Porites* spp. corals (Allison et al. 2024). This seawater temperature rise also increased the contribution of Glx and decreased the contribution of alanine to total skeletal amino acid (Allison et al. 2024). High amino acid and [Asx] were also observed in a *P. australiensis* skeleton deposited during a growth disturbance which was probably associated with high water temperatures (Gupta et al. 2006).

Coral amino acids can be sourced from external seawater, acquired by heterotrophic feeding, or may be synthesised by the coral host or algal symbionts (Ferrier-Pagès et al. 2021). Both glutamic acid and threonine make a significantly higher contribution to total amino acid in the skeletons of Symbiodiniaceae bearing corals compared to non-Symbiodiniaceae bearing corals (Cuif et al. 1999b), which could indicate that these amino acids are primarily sourced from the Symbiodiniaceae. Most glutamate in the sea anemone *Aiptasia pulchella* is derived from algal symbionts (Swanson and Hoegh-Guldberg 1998) in support of this hypothesis. In contrast, alanine and serine make a significantly smaller contribution to skeletal amino acids in Symbiodiniaceae bearing corals compared to non-Symbiodiniaceae bearing corals (Cuif et al. 1999b).

Based on these collective observations, we anticipated an increase in skeletal [amino acid] and shifts in amino acid composition in response to bleaching, but this was not observed. Our samples numbers are limited (six unbleached and four bleached corals), and we note that the p value for this analysis is low (0.07). In addition, the outermost 1 mm of skeleton represents subtly different time periods in each skeleton, depending on skeletal extension rate. Assuming that skeletal extension rates are approximately constant over the year, we estimate that this outermost 1 mm was deposited over periods of ~ 0.8 to 2 months in the bleached corals and 0.5 to 1.2 months in the unbleached corals. The outermost 1 mm may represent a shorter time period, if extension is faster during the warmer, summer months, or a longer time period, if calcification is inhibited by higher temperatures. It is possible that the outermost 1 mm of skeleton includes aragonite deposited before the bleaching event began and any effect of bleaching on skeletal organic matter may be diluted by the inclusion of this pre-bleaching event material in the analysis.

Within these limitations, we observe no significant effect of bleaching on the coral skeletal amino acid concentration or composition of the outermost 1 mm of skeleton. Skeletal amino acids reflect the coral skeletal proteome, which consists of some 30–40 proteins in each of the coral species analysed to date (Peled et al. 2020). Our results suggest that the bleached corals were able to continue to produce the proteins required to maintain skeletogenesis in our study. In unbleached corals, the translocation of photosynthetic products from the Symbiodiniaceae to the coral host contributes significantly to, and can even exceed, the host energetic requirements (Stambler 2011). However, this energy transfer is significantly reduced during bleaching (Palardy et al. 2008). To offset this loss, corals can utilise tissue lipid reserves (Grottoli et al. 2004) or increase heterotrophic feeding (Grottoli et al. 2006). *P. compressa* (a branching species) and *P. lobata* (a massive species) did not increase heterotrophic feeding when bleached (Grottoli et al. 2006; Palardy et al. 2008), suggesting that the use of lipid reserves is more likely in the corals investigated in the present study.

Bleached corals can exhibit reductions in skeletal extension during bleaching (Leder et al. 1991; Allison et al. 1996; Suzuki et al. 2000; Rodrigues and Grottoli 2006), suggesting that loss of Symbiodiniaceae has an immediate effect on calcification. However, on occasion, calcification is reduced after the bleaching event, suggesting that the energetic demands of full skeletogenesis can be met even during bleaching in some corals (Suzuki et al. 2003; Rodrigues and Grottoli 2006).

### Effect of coral bleaching on skeletal aragonite structure

We observe no significant variations in the Raman spectrum ʋ_1_ band FWHM between the bleached and unbleached coral skeletons (Table 1, Fig. 3). The FWHM of this band indicates disorder local to the CO_3_ group in the aragonite lattice and has been linked to the saturation state of the seawater (Ω) from which the aragonite precipitates (DeCarlo et al. 2017), to the inclusion of biomolecules in the aragonite (Kellock et al. 2022; Gardella et al. 2024) and to the precipitation rate of the aragonite (Allison et al. 2024). Increasing Ω and aragonite precipitation rates increases the band FWHM, indicative of increased disorder. Disorder indicates variations of a given ion from its nominal lattice position, and these variations may be more likely to occur during rapid mineral growth. Alternatively the lattice may be distorted by the incorporation of minor and trace ions in place of the mineral host ions, i.e. Ca^2+^ and CO_3_^2−^(DeCarlo et al. 2017; Farfan et al. 2021), and this may occur more frequently during rapid mineral growth (Watson 2004). The inclusion of biomolecules also increases FWHM (and disorder), hypothetically because entrapped biomolecules either distort the lattice structure or influence the incorporation of minor and trace ions (Gardella et al. 2024).

Our data indicate that loss of Symbiodiniaceae has no effect on aragonite lattice disorder in the locality of the CO_3_ group during this bleaching event. Identification of any effect of coral bleaching on the ʋ_1_ band FWHM may be confounded by the interactions of multiple factors which influence FWHM. For example, slow growth rates are associated with reduced FWHM in synthetic aragonite (Allison et al. 2024) but slow growing coral skeletons typically contain increased [amino acid] (Allison et al. 2024) which increases FWHM in synthetic aragonite precipitates (Kellock et al. 2022; Gardella et al. 2024). We note that Farfan et al. (2021) reported a significant difference in the FWHM of slow and fast growing scleractinian corals but no significant relationships are observed between skeletal ʋ_1_ band FWHM and either calcification rate, skeletal [amino acid] or coral calcification media pH in massive *Porites* spp. corals cultured over varying seawater temperature and pCO_2_ (Allison et al. 2024).

Mantanona and DeCarlo (2023) measured the Raman spectrum ʋ_1_ band FWHM in the skeleton deposited by *Porites* spp. corals before and during a bleaching event. Responses were mixed and the FWHM was narrower, wider and unchanged in different specimens during bleaching. On average, the FWHM was 0.04 cm^−1^ broader during the bleaching event compared to before. This is a small mean increase and an increase in this magnitude would not be resolved in the present study.

We do not observe significant relationships between the Raman spectrum ʋ_1_ band FWHM of the coral skeletons and either amino acid content or skeletal linear extension rate (Fig. 4). We note that Raman spectroscopy was conducted over the outermost 200 μm of skeletal material, while the amino acid content was derived from the outermost 1 mm, and skeletal extension is estimated for the 9-month period before coral sampling. These differences in time frame complicate the identification of any relationship between these parameters. However similar relationships are also insignificant in massive *Porites* spp. corals cultured over a range of seawater temperatures and pCO_2_ (Allison et al. 2024).

### The effect of bleaching on skeletal hardness

We observe no significant difference in the Vickers hardness of the outermost 200 µm of the skeletons between the bleached and unbleached corals (Fig. 5). The coral hardness numbers measured here (~ 2.94–3.40 GPa) are broadly comparable to those of other scleractinian corals (Pasquini et al. 2015; Omer et al. 2020; Carrasco-Pena et al. 2020; Moynihan et al. 2021; Tan et al. 2023) even though our measurements were made very close to the skeleton surface, where the skeletal trabeculae are relatively thin. The skeletal trabeculae form from the addition of aragonite at the coral skeletal surface, but are then progressively thickened over several months (Barnes and Lough 1993).

CaCO_3_ biominerals exhibit superior material properties compared to inorganically precipitated analogues (Ghazlan et al. 2021), which is almost certainly, in part, due to the incorporation of protein in the mineral. Hardness was positively correlated with mineral [aspartic acid] and [glycine] in synthetic calcite (Kim et al. 2016). However, [amino acid] in the synthetic calcite were much higher than observed in coral aragonite (Gupta et al. 2006; Kellock et al. 2020; Allison et al. 2024), and it is unclear how [amino acid] influences aragonite hardness. The hardness of *Porites* spp. skeletons, measured by nanoindentation, varied significantly between and sometimes within reef sites and was positively correlated with skeletal density (Moynihan et al. 2021). Skeletal linear extension and density are inversely correlated in many *Porites* spp. corals (Lough and Barnes 2000). In optimal environmental conditions, *Porites* spp. prioritise linear extension over increasing skeletal density (Elizalde-Rendon et al. 2010) resulting in the production of more porous skeleton (Lough and Barnes 2000). Reductions in calcification rate under corals subject to thermal stress (Carricart-Ganivet et al. 2012) may therefore act to promote the skeletal resilience of massive *Porites* spp. corals.

In this study, we identity significant variations in hardness between some individual specimens, but this does not relate to bleaching status (Fig. 5, Supplementary Table S7). The bleached corals analysed here had relatively low skeletal extension rates (Table 1). We did not measure the skeletal density of the outermost layer of the skeleton analysed by microindentation; however, we observe a significant inverse relationship between coral hardness and coral extension rate (in the 9-month period preceding sample collection) (Fig. 6, Table 3). This is suggestive of an effect of skeletal density. We do not observe significant relationships between skeletal Vickers hardness and either skeletal [amino acid] or aragonite Raman ʋ_1_ band FWHM (Fig. 6, Table 3). We found that flaking of the skeletal mount surfaces around the indents occurred significantly more frequently in the mounts of bleached corals compared to unbleached corals (Table S8). Further work could explore how bleaching influence other material properties of coral skeletons, e.g. fracture resistance.

### Implications for coral reefs

We identify no significant effect of bleaching on *Porites lutea* skeleton amino acid composition, aragonite disorder or Vickers hardness. Our sample numbers are small and analysis of a larger sample suite would improve confidence in this conclusion. However, within this limitation, these findings are positive. Many of the bleached corals at this reef sites recovered to normal pigmentation in the weeks following the bleaching event (Allison et al. 1996). Our study suggests that bleaching, from which corals recover, does not significantly affect the aragonite lattice structure or skeletal resilience to plastic deformation. Longer-term bleaching events may have different effects.

Coral skeleton erosion occurs by destructive environmental, chemical, and biological processes (Hernández-Ballesteros et al. 2013). Environmental erosion is promoted by harsh wave action from cyclones and storms, which are predicted to increase in frequency and severity with climate change (IPCC 2022). Biological erosion occurs through physical grazing activities by organisms such as parrotfish and urchins (Cramer et al. 2017), or via chemical attack (Pomponi 1979). We find that coral bleaching, which is survived, does not significantly decrease skeletal hardness. This is a positive finding although we note that skeletal hardness is only one indicator of skeletal resilience, and other metrics (e.g. stiffness and fracture toughness) would give a fuller picture of skeletal resilience to erosion (Fitzer et al. 2019).

Biomolecules, particularly amino acids, are inferred to control the biomineralisation process, as they can influence CaCO_3_ nucleation (Picker et al. 2012), growth rate (Kellock et al. 2020), morphology (Castillo Alvarez et al. 2024), and polymorph (Fang et al. 2023). Our observation of no significant difference in total skeletal amino acid content between the bleached and unbleached coral skeletons (Fig. 2) suggests that coral bleaching has a minimal effect on the skeletal organic matrix and the role that it plays. This is in contrast to ocean acidification and seawater temperature, which have been found to significantly alter the concentrations of amino acids embedded within coral skeletal material (Kellock et al. 2020; Allison et al. 2024).

Finally, we conclude that none of the metrics in our study indicate potential as a skeletal proxy of coral bleaching events. Only 1 coral bleaching event was recorded in the Great Barrier Reef before 1980, while 11 events were recorded between 1980 and 2020 (Erler et al. 2020). While it is certain that coral bleaching events have increased in frequency and severity in recent years (DeCarlo 2020; Babcock et al 2020), it is unclear if events prior to 1980 either did not occur or were not recorded. Identifying a proxy of coral bleaching that could be applied to coral skeletons (which can span up to hundreds of years in age) would therefore be of use. Multiple studies have researched potential proxies (Grottoli et al. 2004; Cantin and Lough 2014; Dishon et al. 2015; Erler et al. 2020) but with limited success. While based on a small samples set, our study indicates that neither skeletal amino acid composition, Raman spectroscopy aragonite ʋ1 band FWHM or Vickers hardness demonstrates potential as a coral bleaching proxy in *Porites* spp.

## Supplementary Information

Below is the link to the electronic supplementary material.

Supplementary file1 (PDF 1364 KB)

## Data Availability

Data are provided within the manuscript or as supplementary information files
