# Supplementary material for: The effects of seawater temperature-induced coral bleaching on the aragonite structure and material properties of massive Porites lutea coral skeletons
Source: Coral Reefs. 2025 Aug 29;44(5):1617–29. doi: 10.1007/s00338-025-02735-5 (PMC12542618; doi:10.1007/s00338-025-02735-5)
Supplement: Supplementary file 1 — Supplementary file1 (PDF 1364 KB) [file 338_2025_2735_MOESM1_ESM.pdf]

The effects of temperature induced coral bleaching on the aragonite structure and material properties of massive *Porites lutea* coral skeletons

Alice Sinclair[1,2], Susan Fitzer[3], Sam Greeves[4], Kirsty Penkman[4], Chalermrat Sangmanee[5], Nicola Allison[1,2]*

1 School of Earth and Environmental Sciences, University of St. Andrews, KY16 9TS, UK.

2 Scottish Oceans Institute, University of St. Andrews, St Andrews, KY16 8LB, UK.

3 Institute of Aquaculture, Faculty of Natural Sciences, University of Stirling, Stirling, FK9 4LA, UK

4 Department of Chemistry, University of York, York, UK.

5 Department of Marine and Coastal Resources, Ministry of Natural Resources and Environment, Thailand

Supplementary data

Figure S1. Raman spectra of analyses focused on coral skeleton and on epoxy resin in the skeletal mounts. a) full spectra and b) magnification from 1060-1160 cm^{-1}. Note the change in intensity on each vertical scale.

Figure S2. Scanning electron micrographs of different indents. Indents were categorised into 4 different groups based on the amount of flaking of skeleton from the mount surface in the locality of the indents. Scar types are defined as: 4 (no flaking of mount surface around scar), 3 (minor flaking of mount surface around scar, 2 (substantial flaking of mount surface but the lengths of the indent are still clear) and 1 (flaking obscures the lengths of the indent).

Table S1. Concentrations of each amino acid in the outermost 1 mm of each coral skeleton and contributions of each amino acid to the total skeletal amino acid. Concentrations are usually means of duplicate analyses and standard deviations are shown. nd= not determined.

Table S2. p values obtained using a Mann Whitney test for equal medians comparing the skeletal amino acid compositions of the outermost 1 mm of skeleton of the unbleached (n=6) and bleached (n=4) corals collected in 1991.

Table S3. Raman spectrum υ_1 band FWHM for all analyses in each coral.

Table S4. Shapiro Wilk test statistic and associated p value testing for normal distributions of the Raman spectrum υ_1 band FWHM dataset and the Vickers hardness dataset within each coral skeleton. A p-value > 0.05 indicates that the data is normally distributed. n.d.= not determined.

Table S5. p values of Tukey's pairwise comparison testing for significant differences in the Raman spectrum υ_1 band FWHM between coral skeletons. Significant differences ($p \leq 0.05$) are highlighted in bold.

Table S6. Vickers hardness and indent category for each indent on each coral skeleton. Indents categorised as type 1 could not be measured as flaking obscured the dimensions of the indent so no hardness is calculated for these indents.

Table S7. p values of Tukey's pairwise comparison testing for significant differences in Vickers hardness between coral skeletons. Significant differences ($p \leq 0.05$) are highlighted in bold.

Table S8. Proportional contribution (as a proportion of 1) of each scar type to the total indentation dataset for each coral skeleton. Scar types are defined as: 4 (no flaking of mount surface around scar), 3 (minor flaking of mount surface around scar, 2 (substantial flaking of mount surface but the lengths of the indent are still clear) and 1 (flaking obscures the lengths of the indent).

Figure S1. Raman spectra of analyses focused on coral skeleton and on epoxy resin in the skeletal mounts. a) full spectra and b) magnification from 1060-1160 cm^{-1}. Note the change in intensity on each vertical scale.

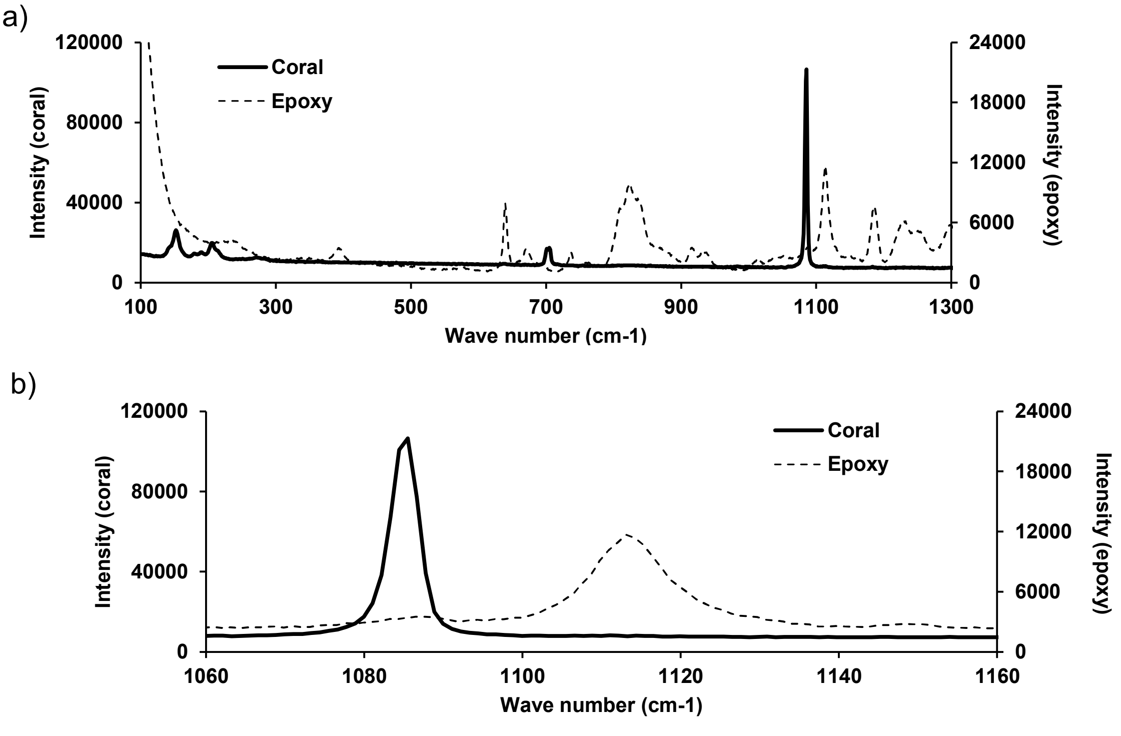

Figure S2. Scanning electron micrographs of different indents. Indents were categorised into 4 different groups based on the amount of flaking of skeleton from the mount surface in the locality of the indents. Scar types are defined as: 4 (no flaking of mount surface around scar), 3 (minor flaking of mount surface around scar, 2 (substantial flaking of mount surface but the lengths of the indent are still clear) and 1 (flaking obscures the lengths of the indent). All scar bars are 5 µm.

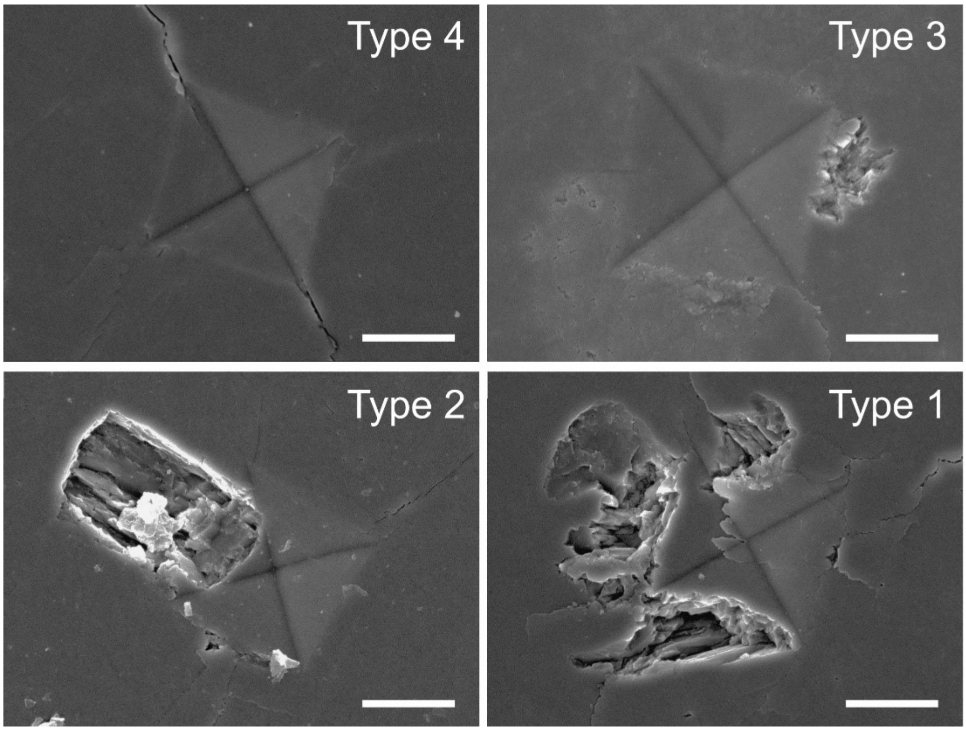

Table S1. Concentrations of each amino acid in the outermost 1 mm of each coral skeleton and contributions of each amino acid to the total skeletal amino acid. Concentrations are usually means of duplicate analyses and standard deviations are shown. nd= not determined.

Sample	Asx	Glx	Serine	L-Threonine	[L-HisL-Histidine]	Glycine	L-Arginine	Alanine	Valine	Phenylalanine	Leucine	Isoleucine	**[Total]**
Mean skeletal [amino acid] pmol/mg													
Unbleached corals													
PB1	643	126	80	50	16	249	26	80	59	44	98	45	**1313**
PB2	762	129	84	49	15	246	19	74	65	42	104	47	**1428**
PB3	761	112	74	42	13	222	14	75	49	37	78	36	**1349**
PB5	896	134	85	45	7	235	19	83	53	39	91	39	**1550**
PB6	804	139	92	50	14	272	21	75	64	39	100	46	**1516**
PB9	1044	177	113	55	15	319	24	89	65	45	88	40	**1885**
Bleached corals													
PB4	1398	237	119	55	11	315	19	102	66	39	92	41	**2309**
PB7	741	150	90	53	11	334	24	86	59	40	90	43	**1538**
PB8	955	125	103	47	9	225	16	89	37	36	77	26	**1597**
PB12	1080	217	105	64	18	366	30	94	75	43	114	53	**2030**

Sample	Asx	Glx	Serine	L-Threonine	[L-HisL-Histidine]	Glycine	L-Arginine	Alanine	Valine	Phenylalanine	Leucine	Isoleucine	**[Total]**
Standard deviation of duplicate analyses of skeletal [amino acid] pmol/mg													
Unbleached corals													
PB1	40	8	4	1	3	86	1	2	0	17	56	7	**40**
PB2	67	14	2	3	8	76	4	3	1	6	54	4	**18**
PB3	73	17	2	1	nd	70	4	1	1	14	46	6	**28**
PB5	57	10	2	0	nd	100	2	0	7	11	58	6	**23**
PB6	58	12	2	0	nd	90	3	2	1	7	53	6	**20**
PB9	54	14	0	1	nd	94	4	0	9	10	51	6	**13**
Bleached corals													
PB4	7	7	2	2	0	90	4	1	2	12	55	6	**72**
PB7	102	23	9	7	2	94	7	8	13	13	48	4	**76**
PB8	48	11	1	3	13	39	3	8	6	14	40	6	**32**
PB12	24	8	1	0	1	70	0	2	3	11	58	8	**43**

Sample	Asx	Glx	Serine	L-Threonine	[L-HisL-Histidine]	Glycine	L-Arginine	Alanine	Valine	Phenylalanine	Leucine	Isoleucine	**[Total]**
Proportional contribution to total skeletal amino acid													
Unbleached corals													
PB1	0.49	0.096	0.061	0.038	0.012	0.19	0.020	0.061	0.045	0.034	0.075	0.034	1.00
PB2	0.53	0.091	0.059	0.034	0.010	0.17	0.014	0.052	0.045	0.029	0.073	0.033	1.00
PB3	0.56	0.083	0.055	0.031	0.010	0.16	0.010	0.055	0.037	0.028	0.058	0.026	1.00
PB5	0.58	0.087	0.055	0.029	0.004	0.15	0.012	0.054	0.034	0.025	0.059	0.025	1.00
PB6	0.53	0.092	0.060	0.033	0.009	0.18	0.014	0.049	0.042	0.026	0.066	0.030	1.00
PB9	0.55	0.094	0.060	0.029	0.008	0.17	0.012	0.047	0.034	0.024	0.046	0.021	1.00
Bleached corals													
PB4	0.61	0.102	0.051	0.024	0.005	0.14	0.008	0.044	0.029	0.017	0.040	0.018	1.00
PB7	0.48	0.098	0.058	0.035	0.007	0.22	0.016	0.056	0.038	0.026	0.059	0.028	1.00
PB8	0.60	0.078	0.064	0.030	0.006	0.14	0.010	0.056	0.023	0.023	0.048	0.016	1.00
PB12	0.53	0.107	0.051	0.031	0.009	0.18	0.015	0.046	0.037	0.021	0.056	0.026	1.00

Table S2. p values obtained using a Mann Whitney test for equal medians comparing the skeletal amino acid compositions of the outermost 1 mm of skeleton of the unbleached (n=6) and bleached (n=4) corals collected in 1991.

Parameter	p value
Total amino acid concentration	0.07
Contribution to total amino acid	
Aspartic acid	0.75
Glutamic acid	0.17
Serine	0.45
L-Threonine	0.83
L-Histidine	0.17
Glycine	0.92
L-Arginine	0.91
Alanine	0.83
Valine	0.20
Phenylalanine	0.11
Leucine	0.13
Iso-leucine	0.20

Table S3. Raman spectrum FWHM of υ_1 band for all analyses in each coral.

Unbleached corals							Bleached corals				
PB1	PB3	PB5	PB2new	PB6	PB2	PB9	PB7	PB12	PB10	PB11	PB8
FWHM	FWHM	FWHM	FWHM	FWHM	FWHM	FWHM	FWHM	FWHM	FWHM	FWHM	FWHM
υ_1 band	υ_1 band	υ_1 band	υ_1 band	υ_1 band	υ_1 band	υ_1 band	υ_1 band	υ_1 band	υ_1 band	υ_1 band	υ_1 band
4.06	4.07	4.04	4.05	4.11	4.19	4.16	4.11	4.14	4.11	4.04	4.10
4.08	4.11	4.13	4.08	4.10	4.15	4.13	4.06	4.07	4.11	4.09	4.09
4.10	4.14	4.15	4.11	4.12	4.17	4.10	4.10	4.12	4.09	4.06	4.10
4.13	4.10	4.07	4.06	4.06	4.18	4.16	4.10	4.10	4.07	4.10	4.10
4.08	4.16	4.18	4.07	4.11	4.14	4.01	4.04	4.16	4.13	4.08	4.12
4.10	4.14	4.13	4.03	4.09	4.13	4.21	3.97	4.17	4.06	4.09	4.18
4.12	4.15	4.12	4.04	4.14	4.03	4.22	4.13	4.03	4.05	4.06	4.07
4.08	4.07	4.14	4.08	4.06	4.04	4.24	4.04	4.05	4.09	4.10	4.09
4.08	4.12	4.12	4.07	4.09	4.08	4.18	4.16	4.06	4.08	4.10	4.16
4.09	4.14	3.98	4.11	4.08	4.09	4.20	4.12	4.09	4.08	4.06	4.18
4.09	4.13	4.01	4.08	4.11	4.14	4.18	4.05	4.08	4.13	4.11	4.13
4.05	4.13	4.09	4.10	4.05	4.11	3.99	4.06	4.07	4.14	4.11	4.16
4.10	4.09	4.02	4.07	4.10	4.16	4.11	4.03	4.13	4.16	4.09	4.12
4.05	4.10	4.04	4.11	4.08	4.12	4.03	4.11	4.09	4.11	4.05	4.15
4.05	4.20	3.99	4.14	4.12	4.14	4.08	4.14	4.16	4.14	4.06	4.13
4.06	4.15	4.09	4.14	4.13	4.12	4.11		4.10	4.11	4.06	4.19
4.07	4.09	4.04		4.12	4.06	4.04		4.12	4.13	4.04	4.11
4.04	4.11	4.10		4.11	4.11	4.15		4.18	4.12		4.05
4.01	4.13	3.98		4.07	4.06	4.13		4.07	4.11		4.15
4.02	4.14	3.99			4.11	4.09		4.01			4.15
4.06	4.14	4.19			4.11	4.18		4.01			4.19
4.04	4.14	4.15			4.11			4.20			4.15
4.06	4.02	4.10						3.95			
		4.09						4.07			
		4.19						4.17			
		4.01						4.14			
		3.98						4.09			
								4.05			
								4.15			
								4.32			
								4.28			
								4.05			
								4.03			
								4.16			

Table S4. Shapiro Wilk test statistic and associated p value testing for normal distributions of the Raman spectrum υ_1 band FWHM dataset and the Vickers hardness dataset within each coral skeleton. A p-value > 0.05 indicates that the data is normally distributed. n.d.= not determined.

	Raman υ_1 band FWHM		Vickers hardness	
Coral Skeleton	Shapiro Wilk test statistic	p value	Shapiro Wilk test statistic	p value
PB1	0.99	0.99	0.98	0.76
PB2	0.96	0.59	n.d.	n.d.
PB3	0.93	0.13	0.99	0.96
PB5	0.94	0.13	0.97	0.68
PB6	0.95	0.48	0.99	0.10
PB7	0.96	0.68	0.94	0.18
PB8	0.96	0.59	0.91	0.068
PB9	0.97	0.76	0.97	0.52
PB12	0.95	0.16	0.94	0.24
PB2new	0.96	0.72	n.d.	n.d.
PB10	0.97	0.76	n.d.	n.d.
PB11	0.94	0.32	n.d.	n.d.

Table S5. p values of Tukey's pairwise comparison testing for significant differences in the Raman spectrum υ_1 band FWHM between coral skeletons. Significant differences ($p \leq 0.05$) are highlighted in bold.

	PB1	PB2	PB3	PB5	PB6	PB9	PB2new	PB10	PB11	PB12	PB7
PB1											
PB2	0.051										
PB3	**0.017**	1.00									
PB5	0.99	0.40	0.21								
PB6	0.79	0.97	0.90	0.99							
PB9	**0.0024**	0.99	0.99	**0.026**	0.56						
PB2new	0.99	0.64	0.43	1.00	1.00	1.00					
PB10	0.34	0.99	0.99	0.87	0.99	0.92	0.96				
PB11	0.99	0.25	0.12	0.99	0.97	0.059	0.99	0.72			
PB12	0.14	0.99	0.99	0.72	0.99	0.86	0.9	1.00	0.51		
PB7	0.99	0.49	0.31	1.00	0.51	0.094	1.00	0.91	1.00	0.79	
PB8	**0.0015**	0.99	0.99	**0.039**	0.57	1.00	0.12	0.90	**0.020**	0.82	0.075

Table S6. Vickers hardness and indent category for each indent on each coral skeleton. Indents categorised as type 1 could not be measured as flaking obscured the dimensions of the indent so no hardness is calculated for these indents.

Unbleached corals										Bleached corals					
PB1		PB3		PB5		PB6		PB9		PB7		PB8		PB12	
VH(GPa)	Category	VH(GPa)	Category	VH(GPa)	Category	VH(GPa)	Category	VH(GPa)	Category	VH(GPa)	Category	VH(GPa)	Category	VH(GPa)	Category
2.89	3	3.27	3		1	3.52	2	3.36	2		1	2.92	2	3.06	4
3.16	3	2.66	2	3.16	3		1	2.52	2	3.56	2	3.77	3	3.29	4
2.90	2	3.62	4	2.26	4	3.68	2	3.64	3	2.96	2	3.94	3	3.21	3
2.86	2	3.33	3	3.89	3	4.02	2	2.63	2	3.94	2	3.41	3	3.92	4
3.24	3	2.92	2	3.36	2	3.18	3	2.70	3	3.36	3	3.75	3	3.66	4
3.56	2		1	2.49	2	3.53	4	2.84	3	3.19	2	3.95	3	2.45	2
3.03	2	3.14	3	3.58	3	2.96	3	3.18	2	3.53	4	3.30	2		1
3.30	3	3.77	4	3.78	3	3.15	2	2.93	2	2.83	3	3.53	2	3.31	2
3.19	2	3.22	2	3.86	3	3.05	2	2.86	2		1	3.79	2	4.14	2
3.29	3	3.49	3	3.86	3	2.69	3	3.35	3	3.60	2	3.60	2	2.98	2
3.37	2	2.69	4	3.19	4	3.66	3	3.44	3	3.67	4		1	3.61	2
	1	2.93	4	3.36	3	3.28	3	3.10	2	3.98	3	3.58	3	3.04	2
3.47	3	2.99	4	3.75	2	2.90	3	3.00	2	3.12	3	3.66	4	3.28	3
3.39	2	3.29	3	3.23	3	3.23	2	2.62	2	2.83	3	3.50	2	2.99	3
3.54	3	2.78	3	2.40	3	2.82	2	3.42	3	3.27	2		1	3.25	3
3.61	2	3.09	3	2.49	3		1	3.27	3	2.81	2	2.43	3		1
3.71	2	2.02	2	3.23	2	3.20	2	3.32	3	2.90	3	3.40	3	2.73	2
3.10	2	2.46	2	3.40	2	2.01	3		1	3.24	2	2.44	3		1
3.75	4	2.67	2		1	2.52	2	3.65	3		1	3.54	2	3.33	3
3.63	4	2.62	2	4.14	3	2.32	2	3.60	3	3.66	3	3.14	3	3.32	4
3.63	3	2.58	2	2.93	3		1	3.10	3		1	3.36	2	3.17	3
	1	2.86	2	2.90	2		1	2.93	3	3.52	2		1	2.34	2
	1	2.97	2	3.50	2	2.63	2	3.17	2	3.01	2	2.84	3	2.49	3
3.85	2	2.44	2	2.99	2		1	1.93	4	3.68	2		1		1
4.16	4	2.65	2		1		1	2.69	3	2.98	2		1	2.55	3
3.99	4	2.88	2	3.22	3	3.08	2	2.89	2		1		1	2.44	2
3.29	2		1	3.53	4	3.36	3	3.44	3				1	2.42	2
2.77	4	2.87	3	3.84	3	2.98	2	3.10	4				1		1
3.72	4	3.27	3	2.81	3	2.93	2	3.50	4			2.89	3		
3.04	2			2.71	2			4.29	2						
3.22	2			2.99	2			2.48	4						
3.84	2			2.97	2			2.75	4						
3.94	4			3.10	2			1.60	4						
3.40	3							4.07	2						
2.93	4														
	1														

Table S7. p values of Tukey's pairwise comparison testing for significant differences in Vickers hardness between coral skeletons. Significant differences ($p \leq 0.05$) are highlighted in bold.

.

Skeleton	*PB1*	*PB3*	*PB5*	*PB6*	*PB9*	*PB12*	*PB7*
PB1							
PB3	**0.0037**						
PB5	0.82	0.25					
PB6	0.15	0.97	0.91				
PB9	0.072	0.96	0.86	1.00			
PB12	0.18	0.95	0.94	1.00	1.00		
PB7	0.10	0.090	1.00	0.63	0.52	0.69	
PB8	0.10	**0.031**	0.96	0.38	0.28	0.44	0.99

Table S8. Proportional contribution (as a proportion of 1) of each scar type to the total indentation dataset for each coral skeleton. Scar types are defined as: 4 (no flaking of mount surface around scar), 3 (minor flaking of mount surface around scar, 2 (substantial flaking of mount surface but the lengths of the indent are still clear) and 1 (flaking obscures the lengths of the indent). p values were obtained using a Mann-Whitney U test for equal medians, comparing the proportion of each scar type to the total dataset between the bleached and unbleached coral skeletons.

Coral	Condition	n	Type 1	Type 2	Type 3	Type 4
PB1	Unbleached	37	0.11	0.41	0.24	0.24
PB3	Unbleached	33	0.06	0.39	0.36	0.18
PB5	Unbleached	33	0.09	0.36	0.45	0.09
PB6	Unbleached	33	0.18	0.52	0.27	0.03
PB9	Unbleached	35	0.03	0.37	0.43	0.17
PB7	Bleached	33	0.21	0.48	0.24	0.06
PB8	Bleached	39	0.23	0.31	0.41	0.05
PB12	Bleached	29	0.21	0.34	0.28	0.17
p value:			**0.04**	0.39	0.57	0.57
